# Chronic pain self-management interventions in primary care – does it make any difference? A qualitative study

**DOI:** 10.1186/s12913-023-09548-8

**Published:** 2023-05-24

**Authors:** Ragnhild Hestmann, Ola Bratås, Kjersti Grønning

**Affiliations:** 1 Department of Public Health and Nursing, Norwegian University of Science and Technology, Postboks 8905, Trondheim, 7491 Norway; 2 Department of Research, Nord-Trøndelag Hospital Trust, Postboks 333, Levanger, 7601 Norway

**Keywords:** Chronic pain, Self-management, Primary health care, Easily accessible, Social support, Qualitative research

## Abstract

**Background:**

Total recovery from chronic pain is difficult. It is therefore important for those who are suffering from chronic pain to find ways to self-manage their pain in daily life. Several chronic pain self-management interventions have been established, but more knowledge is needed to find out what and how it works. This study aimed to explore how the participants in two chronic pain self-management interventions in primary health care experienced the different components of the interventions, and whether the interventions induced any positive changes in the participants’ everyday lives.

**Methods:**

A qualitative study nested within a randomized controlled study using semi-structured individual face-to-face interviews with 17 informants were conducted three months after the interventions. The data were analysed thematically using Systematic Text Condensation.

**Results:**

The main finding was that the informants, from both interventions, self-managed their chronic pain differently in a positive way after they had participated in the self-management interventions. The participants gained new insight from lectures, learning from peers by sharing experiences and belonging to a group, and by recognizing the importance of being physically active.

**Conclusion:**

This study shows that chronic pain self-management interventions consisting of components that learn the participants about chronic pain and include physical activity in a socially supportive environment, may contribute to a positive change in the lives of people living with chronic pain.

## Introduction

Chronic pain is defined as persistent pain with a duration of more than three months. Persistent pain can be malignant, also known as cancer pain, or it can be non-malignant or non-cancer pain [1]. Non-malignant chronic pain due to osteoarthritis, back pain, neck pain, headaches, musculoskeletal disorders, and other conditions, is shown to have an enormous personal and economic burden, affecting more than 30% of people worldwide. The prevalence rate is higher among women and individuals with lower socioeconomic status. Chronic pain is further presented as *“an unpleasant sensory and emotional experience associated with, or resembling that associated with, actual or potential tissue damage”* [2]. It is difficult to achieve total recovery from chronic pain, and people suffering from it must learn to manage and cope with the challenges of having it [3]. Studies show that using a biopsychosocial approach is a valuable framework in the care and treatment of people with chronic pain because the framework takes the “whole person” into account, seeing both the body and mind together as interconnected entities [1, 4].

Both medical and non-medical treatment is used to manage pain. People with non-malignant chronic pain rarely have a long-term effect of medications, e.g., weak, or strong opioids, as opioids-tolerance may lead to increased dosage followed by unwanted side effects. Non-opioid drugs like paracetamol or non-steroidal anti-inflammatory drugs (NSAIDs) might be helpful, but paracetamol alone is often not enough to achieve pain relief and NSAIDs have several side effects. Since it is difficult to achieve total recovery from chronic pain [3], non-medical treatments focusing on function in daily life and well-being [1] is needed.

Much research has been done on non-pharmacological interventions’ effect on people with chronic pain. A recent systematic review showed that non-pharmacological interventions, such as self-management programs, effectively improved physical function and reduced pain among people with chronic pain [5], multimodal rehabilitation showed positive long-term effects on return to work and sick leave [6], and studies on acceptance and commitment therapy (ACT) were effective for daily function, coping, and acceptance of pain [7]. Self-management is defined as “the individual’s ability to manage the symptoms, treatment, physical, psychosocial consequences, and lifestyle changes inherent in living with a long-term disorder” [8]. The aim of self-management programmes for people with chronic pain is often to support the participants on how to manage their pain to improve their health status and quality of life. The programmes often contain several components that are aiming to improve the participants´ self-management of chronic pain in daily life beyond the interventions [9]. The updated best practice for chronic pain management is to develop treatment plans that include establishing a diagnosis, measurable outcomes that focus on improvements in quality-of-life aspects, and using individualized, patient-centred approaches [2]. It is also necessary to have a multidisciplinary perspective in chronic pain management, including physical therapy, exercise, pharmacotherapy, procedural interventions, behavioural treatments, and complementary and integrative therapies. Interventions with a multidisciplinary approach are often delivered to groups of people in different settings and consist of several components that interact with each other, e.g., the content, the participants, and the health professionals who deliver the interventions. Such non-pharmacological interventions are defined as complex interventions and often aim to change behaviours [10]. In addition to evaluating the effect of complex interventions, there is a need for a greater understanding of how and under what circumstances these interventions are effective [11, 12]. The new Medical Research Council (MRC) framework defines evaluation as going beyond asking whether an intervention works (in the sense of achieving its intended outcome), to a broader range of questions including identifying what other impact it has [10]. Qualitative studies of complex non-pharmacological interventions aiming at self-management for patients with chronic diseases, including chronic pain, show that ‘sharing experiences with other patients [13, 14], ‘being in a group’, ‘having a mutual understanding, cohesion, confidence, and openness within the group’ are highlighted as valuable components in the interventions [15]. Other studies have found that the group setting is important for ‘not feeling alone with the pain,’ as it gives the participants a sense of safety and fellowship with others in the same situation [6, 16]. However, even though a group setting is a valuable component, some patients do not find it positive to be in a group with other patients [13, 15]. Another important outcome of non-pharmacological self-management interventions is that the participants gain acceptance of having a chronic condition, where the feeling of being believed is essential [6].

Studies using qualitative and mixed methods designs to gather in-depth knowledge to better understand how interventions contribute to change within their contexts are warranted [10]. This study was nested within a randomized controlled trial (RCT) [17] that found no statistically significant group difference in the primary outcome, patient activation [18]. The RCT was an open, pragmatic, parallel-group RCT, where the participants were randomised to two different non-pharmacological interventions; a self-management course (intervention group) and a low-impact outdoor physical activity (control group). Nonetheless, the study found improvements in experienced pain within both groups in addition to a small statistically significant improvement in global self-rated health in the intervention group [18]. Hence, contextualised understanding of how non-pharmacological self-management interventions induce change, knowledge about what works, how it works, and in which environments, are needed [19] to continue refinements of complex self-management interventions [10]. Against this background, this study aimed to explore how the participants in two non-pharmacological interventions aiming for self-management experienced the components of the interventions, and how the interventions induced changes in their lives with chronic pain.

## Methods

This was a qualitative study nested within a randomized controlled study that was conducted from August 2015 to December 2017 [17]. The data consisted of semi-structured individual face-to-face interviews that were collected approximately 3 months after the participants had attended the interventions.

### Setting

The interventions were delivered in a Healthy Life Centre (HLC) in a major city of approximately 190,000 inhabitants in central Norway. The HLCs are part of Norwegian public primary healthcare services. People can attend HLC activities with or without a referral, in line with the general self-management initiatives increasingly shifting from specialized healthcare services to primary healthcare services in Norway [20]. The participants in the RCT were randomized to a group-based chronic pain self-management course (intervention group) or a group-based drop outdoor physical activity (control group) [18]. The chronic pain self-management course was delivered as 2.5-hour weekly sessions for six weeks comprising theoretical input, movement exercises, group discussions, and sharing experiences among the participants. The drop-in low-impact outdoor physical activity was delivered as a 1-hour weekly session with walking, talking, and simple strength exercises for six weeks. There was no theory in this intervention low-impact outdoor physical activity.

An outline of the interventions is presented in Table 1 below.

**Table 1:** Outline of the interventions

Week	Methodology	Chronic pain self-management course	Drop-in outdoor physical activity
1	Theory	‘What is pain’ ‘The everyday circle’	None
	Physical activity	Movement exercises	‘Walk and talk’+ strength training
2	Theory	‘My challenges’ ‘Problem-solving’	None
	Physical activity	Movement exercises	‘Walk and talk’ + strength training
3	Theory	‘Coping in everyday life ‘Personal qualities and skills	None
	Physical activity	Movement exercises	‘Walk and talk’ + strength training
4	Theory	‘Goal setting’ ‘Action plan’	None
	Physical activity	Movement exercises	‘Walk and talk’ + strength training
5	Theory	‘I can- I have a choice!’ ‘Coping strategies	None
	Physical activity	Movement exercises	‘Walk and talk’ + strength training
6	Theory	‘The way forward ‘Evaluation’	None
	Physical activity	Movement exercises	‘Walk and talk’ + strength training

The theory sessions in the chronic pain self-management course used elements from cognitive behavioural therapy (CBT) and focused on pain theory, barriers in daily life, problem-solving, techniques to deal with being exhausted, poor sleep, and isolation. The participants did the exercise “The ‘everyday life circle” in week 1, consisting of distinct parts to illustrate what a person emphasizes and what the person uses time on [17].

### Informants and recruitment

The informants were recruited by inviting participants that were enrolled in the RCT [17]. The selection of informants was made by consecutively asking participants if they were able to meet at a specific time scheduled for the interviews, i.e., that they wanted to participate in the interview and had the time for it. By asking consecutively, we expected to get sufficient variation in male and female informants. The inclusion criteria were adults of eighteen years or older, self-reported pain for three months or more, and able to participate in group discussions in Norwegian. Exclusion criteria comprised not being able to participate in the outdoor drop-in activity for one hour, pain arising from malignant diseases, and not having the capacity to consent and participate. Recruitment proceeded until no new or relevant data emerged, and the information gathered was found sufficiently saturated for analysis [21].

### Data collection and interview guide

The interviews were conducted by a trained interviewer in a meeting room at the HLC or at the research centre where the interviewer was located. The interviews were audio recorded and transcribed verbatim. Additional notes and reflections were written down immediately after each interview. The transcripts from the first three interviews were read to check if the interview guide needed alterations. The interview guide was semi-structured with open-ended questions to allow the informants to speak freely. The topics were derived from the research question, literature, and the research teams’ clinical experience, comprising experiences with the intervention, whether it was useful, and which parts were useful and why. The informants were also asked about their experiences with the HLC service compared to previous use of other health care services. Moreover, the focus was on coping with chronic pain, if they had experienced any changes in knowledge, use of health resources, and coping ability and mindset. The interviews lasted from 23 to 72 min.

### Data analysis

**Fig. 1:**
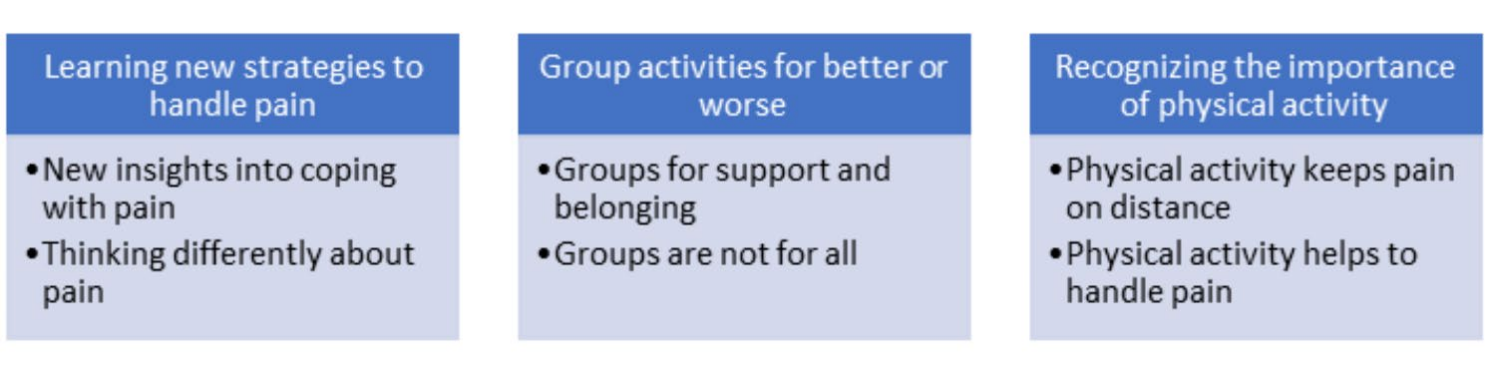
Main themes with belonging subthemes

The data was analysed using Systematic text condensation (STC), a descriptive thematic cross-case analysis strategy based on a phenomenological approach [22]. STC was chosen as it is a structured and well-described step-by-step method for analysis of qualitative data, shown to be suited for presenting experiences as expressed by the informants rather than exploring the possible and underlying meaning of their sayings. The analysis followed the iterative four-step procedure of STC [22]. First, all transcripts were read by all three authors to establish an overview and to gain a general impression of the data, searching for preliminary themes related to the research questions. After reading the transcripts, all authors met to discuss the preliminary themes. Next, the first author systematically reviewed the transcript to identify meaning units representing a different aspect of the informants’ experience with the interventions. Third, the first author classified and sorted the meaning units into code groups, followed by a collective agreement between the authors about the content of the codes. Further, the first author systematically abstracted meaning units within each of the code groups and then sorted them into subgroups. Then, a condensate was abstracted from each subgroup, merging the content from the meaning units of this subgroup. Examples of code groups and subgroups were [1] “Learning and experience things that make me feel better at coping with pain” with the subgroups; “I learned strategies to cope with pain;” and “How I think about pain has changed”, (b) “Group activities are good and not so good,” with the subgroups; “It was supportive to talk to others” and “Experience of belonging to a group”, and c) “Being physically active is essential” with the subgroups; “I learned that physical activity prevents pain” and “I experience that physical activity helps in coping with pain”. After finishing the condensation, illustrative quotations were identified. Finally, the condensed contents were synthesized to generate generalized descriptions and concepts (recontextualized). These are described as the final themes in the presentation of the results (Fig. 1). The research group validated the interpretations and findings against the initial transcripts to ensure that the synthesized result reflected the original context.

### Ethics approval and consent to participate

All informants signed an informed consent form after having received oral and written information to enable them to make an informed choice about participation. The informants were informed that participation in the study was voluntary. The study was approved by the director for health and social affairs in the municipality, and by the Regional Committee for Medical and Health Research Ethics (REK) (2015/ 1030/ REK sørøst).

## Results

A total of 19 study participants were invited to take part in this study and 17 agreed to be interviewed. Table 2 presents the informants’ characteristics, showing a sample of 13 women and 4 men, aged from 32 to 74 years old (mean age 52). The informants suffered from chronic pain due to several causes, but the majority had pain related to musculoskeletal pain, in addition to back pain, fibromyalgia, osteoarthritis, rheumatism, and osteoporosis. Some also had neurological pain, migraine, injuries after treatment, and trauma, and some had pain due to multiple causes. Many of the informants had earlier experiences from participating in different interventions related to coping with pain. Most of the informants were married or had a partner, only three had an education level of four years or more, the majority were on sick leave, disabled/ rehabilitation, or pensioner, and more than half of the informants had experienced a duration of pain for more than 10 years (Table 2).

**Table 2:** Demographic characteristics of the informants

Demographic characteristics	Number
**Gender**:	
Women Men	13 4
**Age**:	
< 35 35–50 51–60 > 60	2 4 6 5
**Marital status**:	
Married/Partner Divorced/Separated Single Widow/Widower	12 3 1 1
**Education level**:	
High school College/University 3 years College/University 4 years or more	6 8 3
**Occupational status**:	
Pensioner Disabled/Rehabilitation Sick leave Working/Education	3 8 3 3
**Duration of pain condition**:	
1–5 years 6–9 years 10 years or more	6 1 10

The main finding was that the informants from both interventions experienced that they self-managed their pain better after attending the interventions. Their experiences of meeting others in the same situation, having the opportunity to share experiences, and belonging to a group were emphasized as important. Informants from both interventions also said that *being physically active* was important for how they handled their pain in their everyday lives. Additionally, some informants from the *chronic pain self-management course* told that they had brought strategies they had learned during the course into their everyday life. The results are further presented as three main themes: (1) Learning new strategies to handle pain, (2) Group activities for better or worse, and (3) Recognizing the importance of physical activity. An overview of the main and sub-themes is presented in Fig. 1. The quotes from the informants are anonymized and marked with gender and type of intervention they participated in, the pain self-management course (course), or the drop-in activity (drop-in) to illustrate the variations of experiences.

### Learning new strategies to handle pain

Several informants explained that they coped better with their pain after the interventions because they related to things they had learned and experienced from the interventions. One informant gave an example of practical coping technique she had learned in the course:

*“I use the breathing techniques that I learned in the course”* (Female, course).

Others described how the lectures, discussions, and homework changed their way of thinking about pain and helped them realize that the pain was harmless. Some reported that they had become physically stronger, they endured more, had better days, and were more open to possibilities on how to think and live with the pain – as stated by the following informant:

*“The stronger I get, the more I endure, and I have learned that pain is harmless”* (Female, drop-in).

Some informants said learning about pain was helpful in managing the pain in daily life, this implied that they were not that exhausted anymore, they were able to contribute to their everyday lives, which made their days more meaningful. Some of the informants also explained that their lives had become better after attending the course because they had learned other ways to cope with their pain.

*“Living with pain is like coping with stress and finding balance, so I feel that the course was very good.”* (Male, course).

Several participants described that their experiences and knowledge from the present and previous interventions also contributed to better coping. They experienced the prior interventions as positive since they learned something new each time, and their experiences and knowledge accumulated.

“*What makes things easier now is the amount of everything I have learned and my experiences, it was positive that I attended that course.*” (Male, course).

*“It meant a lot to meet others in the group, share experiences and learn from each other”* (Female, drop-in).

### New insights into coping with pain

Some informants from the course stated that they learned several practical techniques which helped them to manage their pain in everyday life. The techniques included relaxation, stretching exercises, breathing techniques, and the exercise with the ‘everyday life circle.’ Several also said that it was useful to write down their thoughts and reflections and discuss them with others. The beneficial use of techniques was stated by one informant:

*“Relaxation, breathing and everything from the course is good techniques that I use consciously and brought with me.”* (Male, course).

Another informant explained that he had used the notes from the tasks, homework, and themes discussed in the course to recall what was meaningful for him.

*“I still have the notes from the course and have looked into them several times.”* (Male, course).

Several of the informants said that they had gained new insights into how they could handle their pain by listening to the other participants in the group, and by sharing experiences. They explained getting ideas about physical exercises that could make them feel better and be helpful to ease the pain.

*“Attending more activities and learning from others in a group, I believe in that!”* (Female, drop-in).

*“I learned about the importance of variety in physical exercises from one in the group, who had experience, and that is why I started biking.”* (Male, drop-in).

### Thinking differently about pain

Several of the informants experienced that the tasks from the course made their mindsets focus on other things than pain and that ‘relaxation’ was essential for their mental health.

*“I have learned that relaxation means a lot for the mental part of the pain, and I have managed to see opportunities instead of problems.”* (Male, drop-in).

Some of the informants had learned that when pain is at its peak it will let go, and they tried to be active despite the pain. Others experienced that the pain would go away after exercising, they were not afraid of the pain and felt progress. Several of the informants described that they have learned to do things step by step, to handle commitments without feeling stressed, and to be happy about what they managed to do. One informant felt she had become better at taking responsibility and control of the situation after attending the course.

*“The pain was mine, so I had to stop and deal with it myself – as I felt the course encouraged to.”* (Female, course).

The informants also said that their positive attitude towards life had been reinforced after attending the interventions. They had decided not to get stuck in pain, and not to let the pain identify them. Some experienced that thinking differently about the pain was helpful to keep the mind focused on something else than pain, expressed like this by the following informant:

*“I have learned to cope with pain by using my mind on other things, which has been helpful.”* (Female, drop-in).

The informants’ views of what good health and a good day could be, had been adjusted after participating in the interventions. Having good health was still related to their level of pain, but other issues mattered too. Some said they had learned that it was important to be active, to do something by themselves, and to find motivation to carry on. One described a figure used in the course to see what daily life consists of, called “the everyday circle” as useful in his process.

*“A figure from the course showed how much we emphasized things in life, and for me this was a way of recognizing. When I look at the papers from the course, I can recall the feeling I had about the decision I made about ending work life.”* (Male, course).

### Group activities for better or worse

Most of the informants described the group activities as positive and useful. They reported that a good social life sometimes helps to forget problems. One informant from the drop-in activity said that if the group activity had lasted for a longer period, it might have become a walking group. Several informants said that they still needed group activities to meet others in the same situation and to do activities together. Some of the informants felt lonely and stated that being social helped them mentally.

*“It is important to meet others in the same situation, start talking about how one handles the pain. We managed to add something to each other through discussions”* (Female, drop-in).

The informants told that one of the participants in the drop-in activity initiated a social happening in connection with the drop-in activity, making it possible to get to know each other better. Some of the informants highlighted that their reason for participating in the course was the social aspect of the course and their need to do something regularly. Others stated that they participated in chronic pain interventions to be part of something and for maintaining life quality.

### Groups for support and belonging

Several of the informants appreciated meeting others with similar experiences and problems and several stated that they had missed meeting others in the same situation, and they felt lonely:

*“It was very useful for me to meet others with experience and listen to them, you did not feel so alone.”* (Female, course).

One informant said she had struggled alone in the darkness and experienced interaction with peers as positive and helpful. Others said that their next of kin and family did not understand how they felt. They needed meeting-places for peers who were in similar situations. Some informants had attended several types of non-pharmacological pain management interventions before they participated in one of the interventions in this study. They said that being in a group with others in the same situation was different from other interventions. They also emphasized the importance of the social aspect of the course, especially for those not belonging to working life or having a social network.

*“We were a group, we were peers, and that was most different from other courses.”* (Female, course).

The informants talked about the importance of belonging to something. One informant suggested to organize a group- activity where people who did not attend should be contacted if they did not show up. Then, the participants would experience that they were missed, and that they had some supporters. Another informant emphasized that it was important to consider age and level of functioning of participants when planning group activities since these characteristics influence the group dynamics and benefits.

*“Socially, I would have benefited more if there had been people around the same age and level of functioning.”* (Male, drop-in).

Some informants said that they still had contact with some of the other participants in the group after completing the intervention, and that being in the same group for some time made them feel safe.

*“We sat down at a table outside with coffee and talked, it was nice, and we continued to meet after the activity.”* (Female, drop-in).

### Groups are not for all

The informants experienced the group activities as both positive and negative. Meeting others in the same situation was described as nice, but also a bit scary. As two informants stated:

*“It is a little of both with peers, you might be scared or get motivated to do something.”* (Female, course).

Some had problems identifying themselves with the others, they also mentioned that some participants took too much space and shared too much. Some informants also said that they had nothing in common with the rest of the group. Others expressed that they were not comfortable when some of the participants talked too much about their problems.

*“People want to talk about their problems instead of subject of discussion, I am not comfortable when it is getting out of line.”* (Male, course).

Some informants said it was nice to share experiences with others, but they did not consider it as anything else than talking to ‘someone,’ they did not talk about it as having a therapeutic influence on how they managed their pain.

### Recognizing the importance of physical activity

The informants experienced physical activity as important to manage their pain. Some felt physically stronger after the interventions, and others experienced that being physically active made coping with pain easier.

*“I cope better with pain when being in physical shape.”* (Female, drop-in).

### Physical activity keeps the pain at a distance

One of the informants said she forgot about the problems and the pain when she was biking. Others experienced that going for a walk in the nature or doing exercise felt good, despite having pain. Being physically active and doing positive things, helped the informants to change focus, and not think about the pain all the time.

*“The pain somehow becomes easier when focusing on other things like nature or exercise.” (Female, course)*.

### Physical activity helps to handle pain

The informants agreed that being physically active, exercising and experiencing progression helped them to handle their pain. One informant hoped to get back to work by becoming a better self-manager with more physical activity. Another informant said that being in good physical shape prevented bad periods of pain, and others experienced that being physically active resulted in less pain.

*“Being in good physical shape makes it easier in bad periods, to cope with it and get going.”* (Female, course).

The informants said that the stretching exercises were positive and that feeling stronger was good for their self-esteem.

*“I have learned techniques, such as stretching exercises, to reduce the pain.”* (Male, drop-in).

## Discussion

The aim of this study was to explore how the participants in two non-pharmacological interventions aiming for self-management experienced the components of the interventions, and how the interventions induced changes in their lives with chronic pain.

The main finding was that the informants from both interventions experienced to manage their chronic pain better, after participating in either the chronic pain self-management course or the drop-in outdoor physical activity. These results elaborate the findings from the RCT in which this qualitative study was nested within [18], by explaining how the improvements in both groups were contextualised and expressed. The informants in this study highlighted the importance of meeting others in the same situation and sharing experiences to better self-manage their pain, as other studies also have shown [1, 2, 6] and also confirms the importance of providing people who struggle with chronic pain with sufficient resources to self-manage their pain [3, 19]. In addition, this study provides new knowledge about the fact that a low-cost intervention, such as the drop-in outdoor physical activity offered to the control group, also was helpful for people living with chronic pain. A systematic review focusing on the economic evaluation of self-management interventions in adult chronic patients, in general, found one study where the control intervention was cheaper and more effective than the traditional self-management intervention. In that review, self-management interventions were characterised as multi-faceted programs in which different strategies and techniques were used and applied to help patients to manage their disease over time [23]. The non-pharmacological interventions which the informants in this study attended, had a biopsychosocial approach to chronic pain management [24], acknowledging the importance of treating and caring for people with chronic pain as a “whole” where their bodies and minds are interconnected [1, 4, 5]. The results from this study also show that non-pharmacological interventions aiming for behavioural changes and improvements in quality-of-life aspects, including some sort of physical activity or exercise is good care for people with chronic pain [2]. Since evaluating complex non-pharmacological interventions is challenging [10], this qualitative study shows how the joint components in both interventions; meeting peers, sharing experiences about having chronic pain, belonging to a group, and being physically active were emphasized as important for how the participants handled their pain in their everyday lives. Even though the RCT did not show any statistically significant improvement in self-management measured by the patient activation measure [18], the in-depth qualitative data show that the participants had learned new strategies to handle their pain through the components of walking and talking in the outdoor drop-in activity, the lectures, discussions and homework in the self-management course, and by sharing experiences with peers and recognizing the importance of being physically active to keep the pain at a distance in both interventions.

The findings in this study, therefore, add new knowledge about how and under what circumstances [11, 12] the non-pharmacological interventions induce changes in how participants self-manage their pain. Our findings about the value of being in a group with others, sharing knowledge and experiences to become more open-minded to possibilities in dealing with chronic pain, confirm findings from other studies [13, 14] that these components are important in non-pharmacological interventions for those struggling with a chronic condition. The informants in this study said they learned new techniques to cope with their pain in everyday life. Those who attended the course emphasized ‘relaxation,’ ‘stretching exercises,’ ‘breathing techniques,’ the ‘everyday life circle,’ and ‘writing notes and reflections,’ while those in the outdoor drop-in activity pointed to the variety of physical exercises as important. The findings further showed that by being active, doing something meaningful [25], as well as the relaxation part in the course and ‘the walk and talk’ in the drop-in activity was helpful in changing the mindset and focus on other things than the pain. Other studies have also shown that problem-focused coping, being physically active [26], and feeling empowered are valuable resources for those living with chronic pain [19].

This study further showed that being part of a social community or having a social life was valuable for keeping the pain at a distance, confirming the findings from other studies emphasizing that feeling supported is a vital component in the treatment and care of patients with chronic pain [2, 27, 28]. Some informants in this study were lonely due to varied reasons, e.g., ended work lives. For them was the unformal outdoor drop-in group activity experienced as nice because they met others in the same situation, felt supported and could do activities together with someone. High levels of social support have been shown to have a positive impact on individuals living with chronic pain [27]. Even though the group component is essential in chronic pain self-management, this study also confirms that group activities are not for all [13, 15]. The group setting was sometimes experienced as scary and shocking, showing that some participants may have problems identifying themselves with the group in group-based interventions [13].

Social support seems to be important for maintaining skills for self-management of chronic pain after the intervention, in addition to being in a socially supportive environment while attending an intervention [19]. This study found that some of the informants in the drop-in activity continued to meet after the intervention on their own initiative. This initiative facilitated the need for social support beyond the intervention, illustrating an important message for those in charge of developing and refining non-pharmacological interventions for people with chronic pain. Sometimes, it may be enough to offer a meeting place and let the participants be responsible for how to move forward. The control activity in this study illustrates how a low-cost and less complex intervention in certain environments [19] is feasible and beneficial.

The findings from this study further demonstrate that non-pharmacological interventions in primary care containing the components of lectures (course), discussions with peers (both), physical activity (both) and group activities (both) helped the participants to better self-manage their chronic pain. The results also show that it matters how the groups are assembled regarding age and level of functioning.

Future research should therefore explore the impact of social support from peers beyond non-pharmacological interventions for strengthening the self-management of chronic pain, in addition to assembling the best group composition regarding age, level of functioning, gender and group dynamics.

### Strengths and limitations

This qualitative study used semi-structured face-to-face interviews that allowed the informants to speak freely about their experiences. This provided the opportunity to explore in-depth experiences, arising in another way than in a quantitative study. The interviews were conducted only three months after the interventions, which made it possible for the informants to recall elements from the interventions and whether participating in one of the interventions had any influence on how they self-managed their pain. The data analysis and the results were thoroughly discussed among all the authors. The first author transcribed the interviews, the last author listened to the recordings, and all authors collaborated in analysing the data. Two of the authors were also involved in conducting the RCT which this study was nested within. However, some limitations should be considered. The informants were recruited from two interventions offered at a Healthy Life Centre in a major city in Norway, which is not necessarily representative of all primary healthcare services elsewhere. Anyhow, the sample in this study reflects the population with chronic pain, as there were more women than men in our sample [2]. The informants did also vary in age, duration of pain, education level and occupational status. Further research is however needed to explore if chronic pain self-management interventions make any difference in various health care services in different areas and countries.

## Conclusion

This study shows that non-pharmacological interventions aiming for increased self-management that contain components of physical activity, acquiring knowledge about the impact of chronic pain, and social support, are beneficial for the participants. The participants learned to keep the pain at a distance, and even a very low-cost and little complex intervention made a difference in the everyday lives of people with chronic pain.

Declaration.

## Data Availability

All data supporting the findings in this article is contained within the manuscript.
